# Improving Children’s Social Skills With Virtual Reality: A Tailored Single-Session Training in Special Education

**DOI:** 10.1177/13591045261434617

**Published:** 2026-03-11

**Authors:** Sophie C. Alsem, Anouk van Dijk, Bram O. de Castro

**Affiliations:** 1 Research Institute of Child Development and Education, 1234 University of Amsterdam, Amsterdam, The Netherlands

**Keywords:** social skills, virtual reality, special education, behavior problems, emotional problems

## Abstract

This within-child study examined whether a tailored, single-session virtual reality (VR) training can enhance social skills among children enrolled in special education programs for behavior problems. Forty-nine children (ages 7–13; 85.7% boys; 57.1% ASD, 20.4% ADHD) completed one 20-min VR session. For each child, teachers selected one skill for children to practice with: staying calm (anger regulation), asking to join a group (peer entry), or saying no (assertiveness). Relative to baseline, in-VR observations showed substantial immediate improvements of the targeted skill (η^2^_p_ = .35), but teacher reports two weeks later showed no comparable improvement in the classroom (η^2^_p_ = .07). Teachers also reported no changes in children’s aggressive behavior or emotional problems two weeks later. Children reported moderate levels of immersion and perceived efficacy, low-to-moderate emotional engagement, and high appreciation of the training, VR, and trainer-child relationship. Overall, findings suggest that interactive VR is an attractive tool to practice socially skilled behavior, but that transfer to real life may require more sessions, explicit bridging strategies, and/or more emotionally engaging VR.

## Plain Language Summary

In special education programs for behavior problems, many children find social situations challenging. For example, it can be difficult to stay calm after losing a game, to ask to join a group, or to say no when another child is being pushy. Interactive virtual reality (VR) may offer a safe and fun way to practice these situations with virtual peers. This study examined whether a single session of training in VR may be enough to improve the social skills of 49 children in special education (ages 7-13, mostly boys, many meeting criteria for an autism spectrum disorder or ADHD). For each child, teachers selected one skill for children to practice with: staying calm (anger regulation), asking to join a group (peer entry), or saying no (assertiveness). During the session, children first learned why the skill matters and what they could say or do. Then they practiced the skill three times in VR. For example, a child might practice staying calm while a virtual peer bragged about winning a game against them. Right after the session, children showed clear improvement in the specific skill they practiced, when tested in another VR scenario. However, teachers did not notice changes in childrens socially skilled behavior in class two weeks later, nor in their aggression or emotional problems. Children reported that the training felt somewhat effective. They felt somewhat immersed in the VR world and experienced some emotional involvement. They also reported they really liked the training, the VR activities, and their relationship with the trainer. Overall, interactive VR appears to be a safe and fun way for children to practice social skills. However, to change behavior in the classroom, children may need more than one session, clear strategies to bridge what they learned in VR to their daily life, or VR practice that feels more emotionally engaging.

Children enrolled in special education programs for behavior problems often experience difficulties with social skills (Frostad et al., 2007), including regulating anger, entering peer groups, and responding assertively. Such limited social skills place children at increased risk for various negative outcomes, including aggressive behavior (Glenn et al., 2021) and emotional problems (Obradović et al., 2009). It is therefore important that special education services have tools to improve these skills with their students. Interactive virtual reality (VR)—in which children can practice responding to realistic social situations with virtual peers—may offer this: it can be used flexibly, tailored to individual needs, supports repeated behavioral practice, and is engaging for children (Alsem et al., 2023). This study examined whether such a single-session VR training improves children’s social skills and reduces aggressive and emotional problems.

VR is an emerging and promising intervention method, yet empirical evidence for reducing child mental health problems remains limited (Blanco et al., 2024; Halldorsson et al., 2021). Available evidence suggests that VR can improve children’s social skills (meta-analysis of 15 studies; Zhang et al., 2023), reduce aggressive behavior (one randomized controlled trial; Alsem et al., 2023), and may alleviate emotional problems (systematic scoping review of 11 studies; Blanco et al., 2024). Moreover, single-session VR interventions show initial promise, although this evidence is restricted to anxiety outcomes (Blanco et al., 2024), highlighting the need to examine single-session VR-based social skills training.

VR’s comparative advantage over traditional, typically group-based social skills training may stem from its capacity to immerse children in realistic practice contexts, eliciting the emotions that accompany real-world social interactions and enabling rehearsal under affective load (Alsem et al., 2023). Moreover, children may be motivated to engage with VR, as technology appeals to them (Weisz et al., 2019). Nevertheless, the literature has primarily evaluated multi-session, comprehensive VR intervention packages (Roncero et al., 2025). Such comprehensive intervention packages may not be feasible in an education setting. Evidence of brief, single-session VR training remains limited, despite the practical relevance of flexible application in special education.

This within-child study examined whether a tailored, single-session VR training improved children’s: (a) VR-observed social skills immediately post-training; (b) teacher-rated social skills two weeks post-training; and (c) teacher-rated aggressive behavior and emotional problems two weeks post-training, each compared with pre-training assessments. We also assessed children’s experiences of the training: their appreciation, immersion, emotional engagement, therapeutic relationship, and perceived efficacy. All data, code, and materials (including all items and coding sheets) are publicly available via the Open Science Framework at: https://osf.io/u35jm.

## Method

### Participants

Participants were 49 children (85.7% boys, *M*_age_ = 10.41, *SD* = 1.46) aged 7-13, recruited from two Dutch special-education primary schools serving children who cannot participate in regular education due to severe social, emotional, and/or behavioral difficulties. Teachers reported that most children (71.4%) had a DSM diagnosis, including autism spectrum disorder (57.1%), attention-deficit hyperactivity disorder (20.4%), post-traumatic stress disorder (4.1%), and developmental language disorder (2.0%).

Each school’s special education professional selected classrooms with sufficient time to participate (*k* = 6; *N* = 60). Teachers approached caregivers and children aged ≥12, the majority of whom provided written informed consent (consent rate: 88%). Four children withdrew before training; they did not differ from participants on pre-training teacher-reported social skills, aggressive behavior, or emotional problems, *F* (3, 48) = 0.09, *p* = .963, η^2^ < .01. Power analysis at .80 indicated *N* > 45 to detect small-to-medium within-subject improvements (*g* = .38; Zhang et al., 2023). This study was approved by the University of Amsterdam Ethics Review Board (FMG-8128).

### Procedure

**Figure 1.**
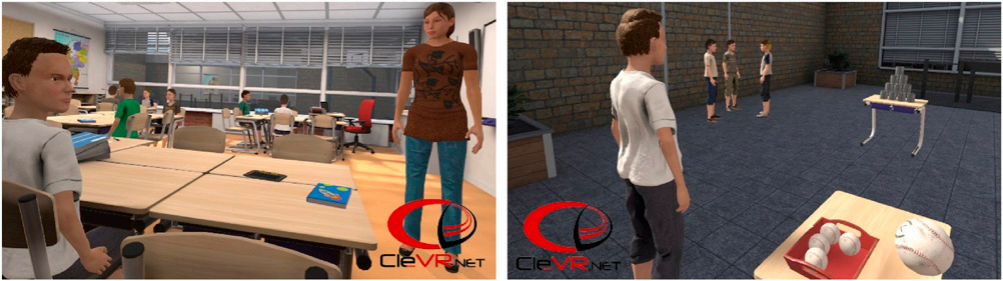
The virtual reality environment

Data were collected at schools in Spring 2024. Teachers completed paper questionnaires rating children’s social skills, aggressive behavior, and emotional problems, both pre-training and two weeks post-training. They also selected one of three social skills that children could practice within VR: anger regulation (*n* = 26), peer entry (*n* = 12), or assertiveness (*n* = 11). Children were individually tested in a quiet room by two of six trained graduate students (one interacting, one observing) in 60-min sessions. The experimenter introduced the VR environment—comprising classroom and schoolyard contexts with experimenter-controlled teachers and peers (Figure 1)—and familiarized children with talking, walking, and grabbing objects (for details on the VR, see Alsem et al., 2023). Children were informed that they could exit the VR at any time, and the experimenter was trained to monitor potential side effects (none occurred, consistent with our previous VR studies). The VR protocol then proceeded as follows: (1) a 5-min pre-training assessment (one scenario); (2) a 20-min social skills training (three scenarios); and (3) a 5-min post-training assessment mirroring the pre-training scenario. Last, children reported their VR training experience.

### Social Skills Training

We developed three tailored, single-session VR trainings targeting anger regulation, peer entry, or assertiveness. These intervention targets were selected by participating teachers and special education professionals using a literature-based list of social skills (Spence, 2003). All trainings were grounded in cognitive-behavioral principles (Marques et al., 2024) and informed by existing interventions (Alsem et al., 2023; Vliek et al., 2019). VR scenarios were developed based on prior pilot work incorporating children’s input (Verhoef et al., 2021) and refined with feedback from special education professionals. For example, an initial anger staircase was replaced with the schools’ emotion pyramid to align with existing practices, resulting in the final training protocols.

The three VR trainings shared an identical structure: First, the experimenter normalized skill practice using a structured worksheet, explained the function of emotions (children drew the target emotion), and introduced a brief cue phrase for the rehearsal of anger regulation (“I’m stepping out for a bit”), peer entry (“Can I join you?”), or assertiveness (“Stop! I don’t like this”). Second, children rehearsed the skill in three VR scenarios (Table S1). For anger regulation, for example, children lost a competitive can-knockdown game to a bragging peer. The experimenter observed and, as needed, coached children (disabling the voice transformer), then provided feedback after each scenario. Children could color a star per scenario to earn a signed diploma. All children received this diploma.

### Measures

#### Social Skills (Observed in Virtual Reality)

We assessed children’s social skills immediately pre- and post-training using standardized, skill-matched scenarios (Table S1). Pre/post scenarios were conceptually similar but perceptually different (e.g., losing different games). Children were asked to treat the virtual school as real and to behave naturally. The experimenter followed scripted interactions and ended each scenario upon a response or after 30 s of nonresponse. The observer recorded children’s verbatim responses and behaviors (see Table S2). After children left, the observer and experimenter independently rated skill-use adequacy on 1-5 scales tailored to the targeted skill (e.g., for anger regulation: 1 = *displays aggressive behavior* and 5 = *uses skill and remains calm*; see Supplemental Material for coding). Interrater reliability was adequate at pre-training (κ = .95) and post-training (κ = .79); discrepancies were resolved by consensus.

#### Social Skills (Teacher-Rated)

We assessed classroom social skills using two items per skill (Alsem et al., 2022; Driestar Onderwijsadvies, 2016). Teachers rated past-week frequency on a scale from 1 (*never*) to 5 (*very often*); for instance, for anger regulation: “This week this student managed to do something about his/her anger” (see Supplemental Material for all items). Item means yielded pre/post scores for anger regulation (α = .80/.86), peer entry (α = .90/.87), and assertiveness (α = .74/.75). We used a variable including only each child’s target skill for the analyses.

#### Aggressive Behavior and Emotional Problems (Teacher-Rated)

We assessed classroom aggressive behavior and emotional problems using three items each (Alsem et al., 2022; Goodman, 1997). Teachers rated past-week frequency on a scale from 1 (*never*) to 5 (*very often*); for instance, for aggressive behavior: “This week this student had a fight with someone” (see Supplemental Material for all items). Item means yielded pre/post scores for aggressive behavior (α = .88/.82) and emotional problems (α = .78/.74).

#### Children’s Virtual Reality Training Experience

We assessed children’s VR training experience using 17 items rated from 1 (*totally disagree*) to 5 (*totally agree*), with mean scores for appreciation (3 items; α = .48; item-level analyses yielded similar results), immersion (4 items; α = .79), emotional engagement (3 items; α = .67), trainer-child relationship (3 items; α = .60), and perceived efficacy (4 items; α = .66; see Supplemental Material for all items). Children also provided overall grades (1–10) for the training and for the VR.

### Analyses

Analyses were conducted in SPSS 29.0. We used four separate repeated-measures ANOVAs to test whether children improved in their social skills (observed and teacher-rated), aggressive behavior, and emotional problems. Effect sizes are reported as partial eta squared (η^2^_p_) and interpreted as small (.01), medium (.06), or large (.14; Cohen, 2013). We summarized children’s VR training experience with descriptive statistics.

## Results

### Data Screening

Table 1 reports descriptive statistics. We found no outliers (i.e., *z* > 3.29). Most variables met normality (*z* < 1.96 for skewness and kurtosis), except observed social skills, for which we used Friedman’s ANOVA. Missing data were minimal (0.8%) and handled via pairwise deletion.

**Table 1.** Descriptive Statistics and Zero-Order Correlations of Pre- and Post-Training Social Skills (Observed and Teacher-Rated) and Teacher-Rated Aggressive Behavior and Emotional Problems

		*M*	*SD*	Range	1	2	3	4	5	6	7
1.	Observed skills (pre)	3.37	1.58	1.00–5.00	-						
2.	Observed skills (post)	4.39	1.12	1.00–5.00	.49^***^	-					
3.	Teacher-rated skills (pre)	2.49	0.90	1.00–4.50	.09	−.03	-				
4.	Teacher-rated skills (post)	2.72	0.95	1.00–5.00	−.09	−.06	.68^***^	-			
5.	Aggressive behavior (pre)	2.50	1.10	1.00–5.00	.11	.11	−.09	−.27^†^	-		
6.	Aggressive behavior (post)	2.61	1.14	1.00–5.00	−.02	−.04	−.12	−.26^†^	.78^***^	-	
7.	Emotional problems (pre)	2.39	1.00	1.00–4.67	−.06	−.14	−.33^*^	−.21	.12	.08	-
8.	Emotional problems (post)	2.51	0.99	1.00–5.00	−.25^†^	−.28^†^	−.31^**^	−.22	.02	.16	.76^***^

*Note.* ^†^*p* < .10. ^*^p < .05. ^**^p < .01. ^***^p < .001.

### Improvements in Children’s Social Functioning

Results indicated large gains in VR-observed social skills, χ^2^ (1) = 19.20, *p* < .001, η^2^_p_ = .35, but not in classroom social skills two weeks later, *F* (1, 47) = 3.42, *p* = .071, η^2^_p_ = .07 (Table 1). Teachers also reported no changes in classroom aggressive behavior, *F* (1, 47) = 0.71, *p* = .404, η^2^_p_ = .02, or emotional problems two weeks later, *F* (1, 47) = 1.25, *p* = .269, η^2^_p_ = .03.

### Children’s Experience of the Virtual Reality Training

Children rated the training highly (*M* = 4.31 out of 5, *SD* = 0.59) and gave high grades to the training (*M* = 8.78 out of 10, *SD* = 1.49) and VR (*M* = 8.45, *SD* = 1.88). Children reported moderate immersion (*M* = 3.33 out of 5, *SD* = 1.01) and perceived efficacy (*M* = 3.54; *SD* = 0.80), and low-to-moderate emotional engagement (*M* = 2.23, *SD* = 1.05). They highly appreciated the trainer-child relationship (*M* = 4.13, *SD* = 0.68).

## Discussion

This within-child study indicates that a single-session, tailored VR training can strengthen children’s targeted social skill when assessed within the training context. However, these immediate gains did not generalize to teacher-reported behavior over two weeks. The large in-VR improvement in children’s social skills aligns with prior work showing that interactive VR can scaffold children’s social behavior and emotion regulation (Alsem et al., 2023; Arts et al., 2025). The lack of classroom change suggests that more is needed to facilitate transfer to everyday settings.

Dose is likely critical: effective social skills and CBT-informed interventions for youth typically provide multiple, spaced opportunities for rehearsal, feedback, and consolidation (Alahmari et al., 2025; Alsem et al., 2023). Yet, additional explanations are possible. Generalization often depends on explicit bridging strategies—such as goal setting, teacher prompts, or reinforcement in class—which were not embedded here. Children also reported low-to-moderate emotional engagement, whereas sufficient engagement is crucial to realistically practice new skills (Sukhodolsky et al., 2016). Although the VR training targeted the teacher-identified priority skill for each child, the VR scenarios were not individualized to children’s daily experiences. Scenarios that more closely mirror children’s everyday struggles may provide a more engaging and relevant practice environment, potentially enabling transfer even after one session. Future research could experimentally manipulate dose, bridging strategies, and scenario tailoring, to identify components that promote generalization.

Methodologically, this study benefitted from standardized VR assessments, strong interrater reliability for VR-observed skills, and skill-tailored protocols. Nonetheless, several limitations warrant caution. First, the absence of a control group precludes causal inference, leaving expectancy effects and measurement reactivity unaddressed. Second, coders of VR-observed skills were not blind to hypotheses (although the verbatim transcripts and behavior logs provide transparent evidence, see Table S2). Third, teacher-reported outcomes comprised only two or three items per construct, and indications of actual aggressive behavior may be relatively infrequent within two weeks; both factors may have limited sensitivity to detect subtle changes. Fourth, although the sample size was sufficient to detect overall effects of the VR training, it was insufficient to compare the effectiveness of the three skill-specific protocols. Last, subtle changes in children’s classroom behavior may not have been detected by teachers; incorporating self- and peer-report measures could provide a more comprehensive and sensitive assessment.

In sum, a single, tailored VR session can yield immediate, context-specific gains in children’s social skills and is well-received by students in special education. To achieve meaningful classroom change and downstream effects on aggression and emotional problems, the training likely requires greater dose, bridging strategies, and/or more personalized, emotionally engaging VR scenarios.

## Supplemental Material


Supplemental Material - Improving Children’s Social Skills With Virtual Reality: A Tailored Single-Session Training in Special Education
Supplemental Material for Improving Children’s Social Skills With Virtual Reality: A Tailored Single-Session Training in Special Education by Sophie C. Alsem, Anouk van Dijk, and Bram O. de Castro in Clinical Child Psychology and Psychiatry.

## Data Availability

The data are available via the Open Science Framework at: https://osf.io/u35jm.
