# Supplementary material for: Improving Children’s Social Skills With Virtual Reality: A Tailored Single-Session Training in Special Education
Source: Clin Child Psychol Psychiatry. 2026 Mar 11;31(3):1025–33. doi: 10.1177/13591045261434617 (PMC13276105; doi:10.1177/13591045261434617)
Supplement: Supplemental Material - Improving Children’s Social Skills With Virtual Reality: A Tailored Single-Session Training in Special Education [file sj-pdf-1-ccp-10.1177_13591045261434617.pdf]

Tables

Table S1

Virtual Scenarios Used for the Social Skills Training (1-3) and Assessments (Pre/Post)

	Anger regulation	Peer entry	Assertiveness
Pre.	Losing a game (block tower), being laughed at	Seeing two peers play (building a block tower)	Being pushed by a peer to give their tablet (math)
1.	Losing a game (throwing at cans), being laughed at	Hearing two peers play “I spy with my little eye”	Being pushed by a peer to make way (block tower)
2.	Being unjustly accused by a peer	Seeing two peers play (throwing at cans)	Being pushed by a peer to do their homework
3.	Being forced to stop playing by the teacher	Hearing two peers play “guess the animal”	Being interrupted while talking with another peer
Post.	Losing a game (throwing at cans), being laughed at	Seeing two peers play (throwing at cans)	Being pushed by a peer to give their tablet (biology)

Table S2

Children’s Verbatim and Behavioral Responses in the Pre- and Post-Training Virtual Scenarios, with Improvement (+), Deterioration (–), or No Change (0) as Determined by Behavior Coding

ID	Pre-training response	Post-training response	Change
Anger regulation			
103	'All falls down', 'uhhh, pfff' looks down and lets shoulders drop.	'Uh oh' and walks through the door.	+
104	Continues building, 'oh no I can catch up'. 'what did you say?' growls a little. Hits the VR child and says 'sorry' after the child said 'ouch'.	Looks around stays calm, 'well played', shouts something unintelligible at the VR child.	+
141	'Oh no' child starts laughing and continues building. Child screams and	Child throws balls and laughs, then walks to the door.	0

ID	Pre-training response	Post-training response	Change
	laughs (cheerful).		
142	'Ah no' 'oh my god' and 'stop I don't like this anymore'.	Child laughs, throws balls and walks away through the door.	+
143	'Wow you are really fast', laughing and pointing 'serves you right'. Knocks over VR child's tower. Laughs and says 'I'm going to knock over your tower' then tries to hit the VR child.	'Yaay I'm the best too, look', keeps throwing. 'Shut up' walks away through the door.	+
144	'Wow your tower is going pretty fast'. Looks at the VR child and throws blocks at the child.	'How did you make this so hard?' 'Yeah done' in a cheerful voice.	0
147	Tries to build and says nothing else.	'Ah almost', then walks out the door.	–
148	'My tower is almost finished too' and 'no way, why are you being so mean?' 'don't laugh at me!'	'Look how fast I am', 'haha look at my balls' and then walks away through the door.	+
150	'Okay', 'that's cheating', 'good job'	Cheating, 'oh no, that's not good' takes ball from VR child.	–
151	Continues building blocks, 'okay', 'what should I do now?'	Keeps throwing cans, looks at VR child and goes through the door and walks away.	0
153	'Oh dear', 'ah it's not so bad that I'm not good at it'.	'I can go even faster', 'ah never mind', walks away through the door.	0
155	Continues building blocks, no further reaction. 'hmm, grr, okay'.	Keeps throwing, walks away through the door.	+
156	Continues building the tower and then destroys the VR child's tower.	Keeps throwing, breathes and groans loudly, keeps going and says nothing.	+
157	Doesn't respond, says nothing and continues throwing blocks. Doesn't respond but knocks over VR child's blocks.	Keeps throwing, gives no reaction. Picks up his ball and throws.	+
169	Continues building, 'grrr' keeps building throws blocks at the VR child.	Throws block at VR child.	0
174	'Wtf', looks at the child. Sighs loudly and	Looks around and stands still 'time isn't	+

ID	Pre-training response	Post-training response	Change
	looks at the child.	as good as here huh' looks around and goes to the door.	
175	Continues building, laughs and looks at the VR child.	Keeps throwing, 'it's only two pins difference'.	0
177	Doesn't respond, then calmly: hey, how then?	Keeps throwing and then opens the door and walks away.	0
183	Keeps building blocks, quickly grabs more blocks and tries to build. Knocks over VR child's tower.	Keeps throwing, takes balls from the VR child and says 'I'm taking your ball haha' and then walks away through the door.	+
184	Continues building blocks and says 'okay'.	Keeps throwing. 'hey I can't knock the last one down' keeps trying to throw.	0
185	'Oh okay', makes a swiping gesture with hand.	'But my balls are gone' then keeps throwing again.	+
188	'He shit, no my tower' child throws blocks at VR child. Knocks over another tower and laughs at VR child.	'Serves you right!' takes ball from VR child. 'you're mean, I'm leaving'. Walks away through the door.	+
200	Looks up, 'what are you talking you still have 3 blocks yeah man what did you say that …. (talking loudly) seriously?!' Walks up to virtual character, says 'good game'.	Silent, sighs deeply, stands still, I'm going to the teacher, this really can't be. This is really not nice.	+
202	Stands up and says softly 'okay'.	Throws ball calmly once more, looks around and opens the door and walks away.	0
204	'What? you're weird', 'can I hit you', 'I want to stick my middle finger up at the child'.	'Yeah I know, high five', 'you're good but shut your m..., high five', 'I'm going to walk away' and walks away through the door.	+
205	'NO!', 'yours fell over too', 'teacher look at this!'	'You're going down Jake'. 'yes won loser', 'teacher, teacher, where's the teacher?', 'I'm leaving', then walks away through the door.	0

ID	Pre-training response	Post-training response	Change
Peer entry			
154	Looks and moves hand a little forward, says nothing else.	'Throwing cans again?', throws ball and hits child.	+
159	Tries to build himself 'I just knocked over your tower'.	'Can I play?'	+
170	'What is this, a music video from 2002?' 'yes I see the tower', 'what is this that I feel now' child touches the real table.	'Hey how old are you', 'what?', 'how did you do that?', 'I'm going to throw this ball at you okay'.	0
173	Looks at VR child walks over and picks up a block, doesn't ask to join.	'Can I join' waits for the answer, then takes the ball and starts throwing.	+
176	No reaction, says nothing and does nothing.	Walks up and asks 'can I join?'	+
179	'Can I join?' starts joining in building blocks.	'Can I join?' child waits for answer and joins in.	0
181	'Hello, may I help build please?' from this moment on playing together.	Walks to the children; 'hello may I please join in?'	0
182	'Yes it's super fun', 'wow very beautiful', 'you are very good at building blocks'.	'Can I play too?' 'yes, thank you'.	+
186	'What?', keeps standing. 'wow super tall' 'it's super tall'.	'Hey, can I join in' and takes a small step towards the other children.	+
189	'Can I join?' waits for response before joining.	'Can I join', waits for the answer and then joins in.	0
191	Laughs and builds without asking.	'Can I join?' and already grabs the ball.	+
206	'Can I help?' Then helps build the tower.	'Hey can I join?'	0
Assertiveness			
101	Says nothing and keeps looking at the table. 'no', 'no'. looks at table.	'No', looks at child. 'no, teacher said not allowed'.	+
145	Looks around and says softly 'okay?', looks ahead and does and says nothing else.	'Okay here then' and gives the tablet to the child.	0
146	'Hmm I don't know', 'yes!' and hands the tablet to the child.	Says loudly 'stop cut it out, I don't like it anymore'.	+

ID	Pre-training response	Post-training response	Change
152	Child lifts up tablet, 'I already had the tablet, I don't know what to do'.	'Oh do you also want to watch a video?' slides iPad towards the other child.	–
158	Looks around, WHAT?! Uhm no unfortunately I'm sorry, I'm sorry but you have to concentrate on the lesson. 'Grrr… But you can have the tablet, but then you're going to do math on it' – gives tablet.	Looks around, 'we can also both watch together?'. 'Hmmm.. uhuhuh I'm going to the teacher if you don't stop now. Then we can watch together okay. That's it. Done.'	+
171	'No' and then again 'no'.	'Nooo' and 'stop quit whining'.	0
172	Looks around and hands the tablet to the child.	'Shh' then says nothing, says softly 'stop I don't like it'.	+
187	'Stop it', 'no', 'shut up', 'are you stupid?', 'shhh'.	'No, because you had to think about trees'. 'shhh, be quiet, stop!'	+
190	Looks at child and holds up the iPad. 'My iPad'. Then hits the child with a book.	'Yeah what is it?', 'are we really going to do this again?', 'go get him' holds the tablet far away from the child.	0
201	Looks around, at student, laughs, holds tablet and says 'no'.	'No the teacher said not allowed, here you can have this book' – gives book.	0
203	Says softly 'Oh, but I have to study explanation', 'I can't, because I have to study explanation'	'I can't, you're confusing me.' 'No,no,no, please don't do it.' 'Because it's actually not allowed.' 'Why do I have to give it if eh.. please stop I don't like it anymore.' 'Teacher, I watched the video!' 'Teacher she's being annoying, because when I was watching a video she asked can I have the tablet.'	+

Coding System for VR-Observed Social Skills

These categories were used to assess the adequacy of children's responses in virtual reality:

Anger Regulation

The child regulates their anger by walking away from the situation.

1. No, the child reacts angrily toward the peer (e.g., tries to hit, uses name-calling or mean language toward classmates, yells that they do not like it).
2. The child reacts irritated/angry toward the peer but without verbal or physical aggression; the child does not do anything to regulate the anger.
3. The child shows visible irritation, remains in the situation, but does not react inappropriately toward the peer. The irritation/anger does not decrease during the situation. OR the child becomes visibly irritated and attempts to regulate their anger, but the strategy is not effective (e.g., shouting or cursing while walking away).
4. The child is noticeably angry (e.g., growling, breathing heavily, or saying they don't like how the peer is acting) and uses a strategy to reduce their anger. The anger remains visible, but no inappropriate behaviors are shown.
5. Yes, the child remains calm or calms themselves down after showing initial signs of irritation—for example, by walking away.

Peer Entry

The child asks to join in play.

1. No, the child stands still and does not say anything to the peers.
2. No, the child walks over to the peers but does not ask to join in OR the child joins the game without saying anything OR the child says something like "I'm playing too" but does not actually ask.
3. The child makes contact with the peers by commenting on the game, but does not ask to join.
4. The child walks over and asks to join the game, but doesn't wait for a response and starts playing immediately.
5. Yes, the child walks over and calmly asks if they can join, waits for the answer, and then joins the game.

Assertiveness

The child indicates that they are not going to give the tablet.

1. No, the child gives the tablet to the peer when asked.
2. No, the child does not indicate that they won’t give the tablet / says nothing.
3. The child tries to gently indicate that they don’t want to (or are not allowed to) lend the tablet, but it doesn’t come across clearly.
4. Yes, the child gently indicates that they are not going to give the tablet. They seem somewhat unsure when setting their boundary (e.g., by speaking softly, looking down, or struggling to find the right words).
5. Yes, the child clearly states that they are not going to give the tablet. They sound confident when setting their boundary (e.g., by speaking clearly, making eye contact, and expressing themselves well).

Items of Teacher-Rated Outcomes

Social-Emotional Skills

Items are rated as: 1 = *never*, 2 = *rarely*, 3 = *sometimes*, 4 = *often*, 5 = *very often*.

1. This week... this student told other children what he/she thought about something
2. This week... this student was so angry that he/she couldn’t stop him/herself anymore
3. This week... this student asked other children if he/she could join in
4. This week... this student was able to stand up for him/herself
5. This week... this student managed to do something about his/her anger
6. This week... this student asked other children to play together

Items are averaged to create scores for:

- anger regulation (items 2 reversed and 5)
- peer entry (items 3 and 6)
- assertiveness (items 1 and 4)

Aggressive Behavior and Emotional Problems

Items are rated as: 1 = *never*, 2 = *rarely*, 3 = *sometimes*, 4 = *often*, 5 = *very often*.

1. This week... this student had a fight with someone
2. This week... this student had many fears, was easily scared
3. This week... this student kicked or hit someone
4. This week... this student was often unhappy, down-hearted or tearful
5. This week... this student called someone names
6. This week... this student had many worries, often seemed worried

Items are averaged to create scores for:

- aggressive behavior (items 1, 3, and 5)
- emotional problems (items 2, 4, and 6)

Items of Children's VR Training Experience

Items are rated as: 1 = *totally disagree*, 2 = *disagree*, 3 = *a little*, 4 = *agree*, 5 = *totally agree*.

1. I enjoyed taking part
2. I got completely absorbed in the virtual reality
3. I didn't find taking part today useful
4. Some things in the virtual reality really made me a bit angry
5. Taking part today helped me
6. The researcher listened to me well
7. The virtual reality felt real
8. I thought the practice today was boring
9. I never felt angry in the virtual reality
10. The researcher understood me well
11. I learned a lot from the practice today
12. It felt like the virtual reality was really happening to me
13. The practice today didn't really work for me
14. Sometimes I felt a bit angry in the virtual reality
15. I would recommend taking part to other children
16. During the virtual reality, it felt like I was really experiencing things
17. I felt comfortable with the researcher

Items are averaged to create scores for:

- appreciation (items 1, 8 reversed, and 15)
- immersion (items 2, 7, 12 and 16)
- emotional engagement (items 4, 9 reversed, and 14)
- therapeutic relationship (items 6, 10, and 17)
- perceived efficacy (items 3 reversed, 5, 11, and 13 reversed)

Last, we asked children to provide an overall grade:

- If I had to give a score from 1 to 10, I would give taking part in the training a...
- If I had to give a score from 1 to 10, I would give practicing in the virtual reality a...
